# Bilingualism as a risk factor for false reports of stuttering in the Early Childhood Longitudinal Study (ECLS-K:2011)

**DOI:** 10.3389/fpsyg.2023.1155895

**Published:** 2023-07-20

**Authors:** Susanne Gahl

**Affiliations:** Department of Linguistics, University of California at Berkeley, Berkeley, CA, United States

**Keywords:** stuttering, bilingualism, school-age children, linguistic minorities, parent report, epidemiology, age factors, sex factors

## Abstract

**Introduction:**

Bilingualism has historically been claimed to be a risk factor for developmental stuttering. The Early Childhood Longitudinal Study, Kindergarten Class of 2010-11 (ECLS-K:2011) ostensibly contains evidence to test that claim.

**Methods:**

We analyze data from monolingual and bilingual children in Kindergarten through fifth grade in the ECLS-K:2011.

**Results and discussion:**

The prevalence, male/female ratio, and onset and recovery of reported stuttering in the ECLS are inconsistent with widely-accepted clinical reports of stuttering. We argue that the reported figures may be misleading. We discuss some factors that may inflate the reported prevalence, including a lack of awareness of the difference between stuttering vs. normal disfluencies, and the informal usage of the word “stuttering” on the part of teachers and parents to describe typical disfluencies.

## Introduction

Population-based surveys constitute an important source of information about demographic trends in the distribution of disabilities, and about psychosocial, cognitive, and other variables of interest to a wide range of research endeavors in Psychology, Speech and Hearing Sciences, and related fields (see e.g., Zablotsky et al., 2019). Research on stuttering has been informed by large-scale surveys since at least the beginning of the twentieth century (see e.g., Conradi, 1912 for an early overview).

Stuttering is a neurodevelopmental disorder whose symptoms include involuntary sound prolongations, repetitions, and silent “blocks” (or “tense pauses”). These symptoms differ from non-stuttering-like (or “typical”) disfluencies, i.e., disfluencies not indicative of any disorder, such as unfinished words (or “word fragments”, such as *I was riding my scoo.. bicycle*), phrase repetitions (*I saw a huge, a huge..*.), interjections (*um, uh, ‘kay*), and revisions (e.g., *My brother...sister also went*). Stuttering is a multifactorial disorder with genetic, neurophysiological, emotional and behavioral causes (see e.g., Smith and Weber, 2017 for discussion). Socioemotional and cognitive development, comorbidities, and demographic factors of stuttering are topics that have unquestionably benefited from analyses of population-based samples. For example, Boyle et al. (2011) analyzed the prevalence of a range of disabilities, including stuttering, across different socioeconomic groups, based on the National Institutes of Health National Health Interview Survey spanning the years 1997–2008 (NHIS, Centers for Disease Control and Prevention, 2019). Briley and Merlo (2020) investigated the interaction of stuttering with insomnia and allergies, using NHIS data for the year 2012. Another example is Choo et al. (2020), who analyzed comorbities, socioemotional, and cognitive development among children who do vs. do not stutter, based on NHIS data from 2006 to 2018. These population-level studies with broad coverage of demographic variables have the potential to complement analyses of databases specifically designed for stuttering research, such as Yairi and Ambrose (1999), Brundage et al. (2016), Rocha et al. (2019), and Walsh et al. (2021). Taken together, clinical samples and population-level data enable research that neither can support on its own.

The interaction of bilingualism and stuttering is a topic of long-standing debates and ongoing research that could potentially benefit from population-based research (see e.g., Van Borsel et al., 2001; Van Borsel, 2011; Byrd et al., 2015a; Choo and Smith, 2020; Chaudhary et al., 2021). As we review in more detail below, the question whether bilingualism constitutes a risk factor for stuttering has a long history and a slim evidence base. In fact, there are numerous gaps in research on stuttering in bilingual children, as pointed out, for example, in Kohnert and Medina (2009), Brundage et al. (2016), and Choo and Smith (2020). Researchers in this area are bound to welcome any reputable and substantial source of data. The scarcity of data and the sense that more information is urgently needed make it especially important to check the validity of any sources that do come along: Lack of information impedes checks and balances.

The NHIS, i.e., the source investigated in the studies mentioned above, unfortunately does not include information about child bilingualism. A population-level database that does include this information is the Early Childhood Longitudinal Study, Kindergarten Class of 2010-11 (ECLS-K:2011) (Tourangeau et al., 2019). The ECLS-K:2011 (henceforth “ECLS”) followed a nationally representative sample of roughly 18,000 children through their elementary school years. The ECLS includes information about socioeconomic factors, race/ethnicity, children's health, as well as languages spoken in the child's home, collected in interviews with parents, teachers, and children. The database has enabled research on child development, education, and learning, the roles played by socioeconomic status, and race and ethnicity; for a summary of the history and design of the database, and an overview of some of its applications, see West (2017).

The ECLS ostensibly includes information about stuttering. Unfortunately, we see reason to believe that the information might be misleading and might not actually reflect stuttering at all. Crucially, and unlike in the NHIS, parents were not asked whether a formal diagnosis of stuttering had been made, but only whether their children had “a problem with stuttering”. No indication was given that identification of stuttering might call for formal assessment, and no definition of stuttering was provided.

The lack of any clarification as to what was being asked is particularly problematic given the use of the word “stutter” outside of clinical contexts to mean “to speak haltingly”. Although that usage is deeply troubling to many people who stutter and their allies, it is common and, consequently, continues to be propagated by dictionaries. For example, the first definition of the verb “to stutter” in the Oxford English Dictionary (OED Online, 1989) is “To speak with continued involuntary repetition of sounds or syllables, owing to excitement, fear, or constitutional nervous defect”. No clarification was included in the question as to whether the word “stuttering” in the ECLS was intended as the name of a disorder. Starting in the third-grade round of data collection, a “help text” was available for interviewers to read to parents upon request. The help text read “*A Stuttering problem refers to difficulty producing fluent (flowing or effortless) speech. The child cannot speak sentences or groups of words with ease. Instead, the child hesitates before saying some words. They may seem as if they are mentally blocking on a word. This hesitation makes it hard for the listener to understand what the child is trying to say*.” That explanation falls short in several respects: Most children who stutter produce sentences or groups of words with ease some of the time. Most importantly, the help text fails to differentiate stuttering from normal disfluencies, tip-of-the-tongue states, or other speech hesitations not due to stuttering, and it falsely implies that symptoms of stuttering generally impede intelligibility.

We see reasons to think that bilingual children may be particularly liable to be described informally by teachers and parents as “stuttering”. First, bilingual children who are English Language Learners may speak non-fluently if their proficiency in the language of the classroom is low. Secondly, speech characteristics seen in (non-stuttering) bilingual speakers, regardless of proficiency, have been found to overlap with disfluencies considered in stuttering diagnosis (Byrd et al., 2020), possibly to the point of exceeding diagnostic threshold. Specifically, it has been claimed that speakers of more than one language produce what are sometimes known as “mazes” at higher rates than monolingual speakers. Mazes are “disruptions in the forward flow of speech that are characterized by the production of a string of words, initial parts, or unattached fragments of words” (Byrd, 2018, p. 325) (e.g., *He went looking for....looking for his frog; He climbed a ladd.. out of the jar*). Mazes can arise as part of self-correction (“revision”) and other typical disfluences. Importantly, mazes include repetitions of sounds, syllables, and monosyllabic words (e.g., *The b..boy lost his frog; he... he... he... he didn't find him*.) Maze production may make it more likely that bilingual children will be perceived as disfluent and, possibly, described informally as “stuttering”. In fact, there is reason to think that stuttering bilingual children are at risk of both overdiagnosis and underdiagnosis of stuttering, a point we review in more detail below. For these reasons, we investigated whether the ECLS was a reliable source of information about stuttering. We hypothesized that it might not be, and especially so for bilingual children. Although there are many open questions about stuttering, there are also some widely accepted findings. We reasoned that, to the extent that the ECLS data are inconsistent with well-established findings, they were unlikely to reflect stuttering. If the degree of inconsistency is substantially higher for bilingual groups compared to monolingual ones, this would imply that bilingualism puts children at risk of being falsely reported to stutter in the ECLS.

Three sets of findings in particular can be said to approach the status of consensus findings in stuttering research. These are (1) rough estimates of overall prevalence, particularly past early childhood, (2) estimates of the male/female ratio of stuttering, and (3) the age ranges during which onset and recovery are most likely to occur. It should be noted that there are ongoing disagreements over classification and identification of cases and symptoms that affect even the most general observations about prevalence, recovery, and diagnostic criteria (see e.g., Einarsdóttir and Ingham, 2005; Brocklehurst, 2013; Howell, 2013; Yairi, 2013; Byrd, 2018 for discussion). In the current paper, we use the estimates identified in Bloodstein et al. (2021) as reference points, without intending to imply complete agreement on any of these points.

We ask whether the ECLS data ostensibly about stuttering are broadly consistent with widely cited clinical reports. If the reported rates reflect actual stuttering, they should be similar to prior observations. If, on the other hand, the figures reported in the ECLS diverge markedly from those reported elsewhere, then that would be an indication that the ECLS data reflect factors other than stuttering.

Given the enormous potential value of the ECLS, it is important to establish whether the information in it actually reflects stuttering. To that end, we asked whether the ECLS data were plausible, in light of clinical research on stuttering.

We asked the following questions:

**Q1** Does the proportion of children reported to stutter differ depending on whether another language besides English is spoken at the child's home?
**Q2** What is the male/female ratio among children who reportedly stutter? Is that ratio plausible, and does it depend on the child's home language?
**Q3** Are the patterns of persistence of and recovery from (supposed) stuttering consistent with clinical research?

## Background

### Clinical findings on onset, prevalence, persistence, and male/female ratio of stuttering

#### Age of onset and recovery

The childhood incidence of stuttering is thought to be between 5 and 10 percent (Yairi and Ambrose, 2013; Bloodstein et al., 2021). Recovery, with or without intervention, is thought to occur in approximately 80% of children who stutter (Bloodstein et al., 2021). The adult prevalence is usually thought to be about 1% (Yairi and Ambrose, 2013; Bloodstein et al., 2021).

Both onset and recovery are most commonly observed before the early elementary school years (Yairi and Ambrose, 2013; Franken et al., 2018). Yairi et al. (2005), cited in Yairi and Ambrose (2013), one of the most extensive available studies of stuttering in English-speaking children, found that 95% of onsets in their sample occurred by the fourth birthday. Yairi and Ambrose (2013) further note that recovery most often occurs before age 5–6, i.e., the typical age when children enter elementary school in the U.S.. Consistent with these observations, Bloodstein et al. (2021) note that most published reports find that stuttering prevalence does not appear to change much over the course of the elementary school grades, although some children do recover during those years. At least one population-level study is consistent with a scenario in which few new cases of stuttering emerge during the elementary school years: Zablotsky et al. (2019) found (based on the 2009 to 2017 National Health Interview Survey) that stuttering prevalence decreased from 2.73% in the youngest age group considered (ages 3–5), to 2.26% for the next-older group (ages 6–11), a difference that reached statistical significance. Parents were asked whether their children had “had stuttering or stammering during the past 12 months”. The ages in the two groups that were compared therefore ranged from 2–5 and 5–11, respectively. Zablotsky et al. (2019) found that the prevalence was also significantly lower (at 1.43%) in the oldest group, aged 12–17 than in the group aged 5–11. That latter observation may reflect recovery during the elementary and middle school years.

In summary, most children reported to stutter in Kindergarten (if they in fact have the disorder) would be expected to still stutter in the later grades. Conversely, we would expect few children to stutter in later grades who were not also reported to stutter earlier on. We should also expect little change in stuttering prevalence after grade 1 in the ECLS.

#### Male/female ratio

Stuttering is more common in boys than in girls, and the male/female ratio increases with time since onset (and hence, age), a pattern usually attributed to recovery being more likely in girls than in boys (see e.g., Yairi and Ambrose, 1999, cited in Yairi and Ambrose, 2013). Among preschool children, male/female ratios have been found to be as low as 1.5:1 (in the studies reviewed in Bloodstein and Bernstein Ratner, 2008; Bloodstein et al., 2021). By the end of elementary school, the male/female ratio is estimated to be as high as 3:1 or 4:1, i.e., similar to the rate of 4:1 usually given for adults (Craig and Tran, 2005; Yairi and Ambrose, 2013; Bloodstein et al., 2021). Consistent with this, Choo et al. (2020) found a male/female ratio of 2.5:1 in a sample spanning ages 3-17 (the National Health Interview Survey, 2006 to 2018). On the basis of data in Yairi et al. (2005), Smith and Weber (2017) estimate that the probability of eventual recovery is about 0.4 for children who stutter around the fifth birthday, decreases rapidly during the sixth year of life, and approaches zero around the seventh birthday. On the basis of these figures, we should expect the male/female ratio in Spring of Kindergarten (the earliest time when the question about stuttering was asked in the ECLS), and in grade 1, to be perhaps somewhat higher than that typically observed for the preschool years. The direction of change, to the extent that there is a change, should move the ratio closer to the 4:1 ratio seen in adults.

### Bilingualism as a claimed risk factor for stuttering

The claim that bilingualism might be a risk factor for stuttering has a long history, itself part of a broader history of comparisons of stuttering prevalence across societies, and across racial, ethnic, socioeconomic, and linguistic groups. Relevant empirical evidence is sparse, however. By far the most frequently cited study comparing stuttering prevalence in monolingual and bilingual groups is Travis et al. (1937). Travis et al. (1937) reported stuttering prevalence to be higher in bilingual (and trilingual) schoolchildren, compared to monolingual ones. Travis et al.'s (1937) study has been severely criticized on methodological grounds (see e.g., Byrd et al., 2015a; Choo and Smith, 2020; Bloodstein et al., 2021 for discussion), and the reported figures have been argued to be internally inconsistent (Gahl, 2020).

More recent studies have found stuttering prevalence in bilingual groups to be no different from that in monolingual groups. For example, Mohammadi et al. (2008, 2010) found no evidence suggesting a higher stuttering prevalence among Kurdish-Farsi bilingual children in Iran, compared to monolingual Farsi-speaking children.

On the other hand, Howell et al. (2009) reported some evidence consistent with the notion that early bilingualism might be a risk factor for stuttering. Howell et al. (2009) examined a sample of children who had been referred to stuttering clinics in the UK. It was found that fewer children stuttered who exclusively used a “minority” language, i.e., a parental language other than English spoken in an immigrant community, until school entry, compared to children who used both their parental languages and English before school entry. In a control group of children who did not stutter, the proportion of children who were monolingual in a language other than English until school entry vs. bilingual was reversed. Howell et al. (2009) also found that the proportion of children who used both their parental language and the majority language (English) vs. those who learned English only after school entry was higher among the children who stuttered, whereas these proportions were reversed in a comparison group of bilingual children who did not stutter (age-matched and from similar socioeconomic backgrounds as the children who stuttered). Howell et al. (2009) concluded that parents' use of a language spoken by a linguistic minority alongside English before school entry was associated with a higher risk of stuttering (see Byrd et al., 2015a and Choo and Smith, 2020 for discussion).

Theoretical frameworks that have been cited in support of the claim that bilingualism might be a risk factor for stuttering are the “demand and capacities” model (Andrews et al., 1983; Adams, 1990; Starkweather and Gottwald, 1990), appealed to in Karniol (1992), and the Stuttering as Suprasegmental Sentence Plan Alignment model, proposed in Karniol (1995). It should be noted that Karniol (1992) and Karniol (1995) are based on a single-case study of a child who followed a fairly typical trajectory of onset around 26 months years of age, and recovery at 31 months. Although onset and recovery coincided with changes in the child's use of two languages, it is not clear whether any particular explanation is needed for onset and recovery to occur at these points. These studies have nevertheless been influential, as rare attempts to put bilingualism as a risk factor on a theoretical footing.

Theories about causes and mechanisms of stuttering outline some potential mechanisms by which bilingualism might affect if not the prevalence of stuttering, then the frequency and location of stuttering events within utterances in individuals who do stutter (see Choo and Smith, 2020; Brundage and Ratner, 2022 for recent reviews). Cognitive functions through which bilingualism may interact with symptoms of stuttering include both those that are specifically involved in language processing, such as grammatical and lexical knowledge, as well as domain-general cognitive functions, such as Executive Function.

### Diagnosis and misdiagnosis of stuttering

Any discussion of prevalence estimates, onset and recovery, and risk factors of stuttering is complicated by disagreement over what the diagnostic criteria for stuttering should be, and even how stuttering should be defined (cf. Tichenor and Yaruss, 2019). Moreover, decisions about intervention and treatment depend on additional factors besides diagnosing the presence of the disorder, such as severity, life impact, and attitudes toward stuttering. Instruments for assessing such factors in speakers of English include the SSI-IV (Riley and Bakker, 2009, for assessing behavioral severity), OASES (Yaruss and Quesal, 2008, for ascertaining perceived life impact), and the UTBAS (Iverach et al., 2015, for assessing attitudes and beliefs). As Cavenagh et al. (2015, p. 163) point out, “[s]imply listing symptoms relating to speech performance as a method of identifying stuttering in children is problematic”. Despite this, it appears to be the case that “the majority of definitions of stuttering that are available in the research and clinical literature focus on overt speech symptoms” (Cavenagh et al., 2015, p. 163), i.e., setting aside self-report, secondary stuttering behaviors, first-person perception of stuttering and socioemotional impact. The diagnostic process that might take place in a therapy setting is not always the basis for classifying participants as stuttering vs. not in research literature.

Different diagnostic criteria result in different diagnoses and hence, different estimates of stuttering prevalence and severity Franken et al. (2018), for example, compared two sets of criteria. By one criterion, each participant was classified as “a child who stutters” if the child produced 3 or more stuttering-like disfluencies per 100 syllables, following Yairi and Ambrose (1999) or if the child's SSI-4 score was 9 or higher. An extended set of criteria took children's self-report into account. Franken et al. (2018) argue that recovery status can be determined more accurately if self-report is taken into account.

Stuttering diagnosis purely on the basis of the frequency and types of disfluencies is particularly problematic for populations whose speech characteristics overlap with symptoms of stuttering. Bilingual children appear to be one such population: Byrd et al. (2015a) argue that certain speech characteristics of bilingual children who did not stutter put them at risk of false positive diagnosis. Byrd et al. (2015a) found that the rate of stuttering-like disfluencies in speech samples from bilingual children who did not stutter exceeded a common diagnostic threshold. As Byrd (2018) put it, with reference to the diagnostic threshold of 3 or more stuttering-like disfluencies per 100 syllables, “(...) if the 3% guideline had been employed, 100% of these bilingual children would have been classified as children who stutter even though there was no concern on the part of child, parents, teachers, or clinicians regarding their fluency.” Byrd et al. (2016, p. 1) conclude that “bilingual children are not at increased risk for development of stuttering, but they do appear to be at increased risk for false positive diagnosis of stuttering”.

In fact, there is evidence suggesting that bilingual children are at risk of both overdiagnosis and underdiagnosis of stuttering. Byrd et al. (2015b) asked 14 trained Spanish-English bilingual speech-language pathologists (SLPs) to listen to English and Spanish speech samples produced by two bilingual children matched for age, gender, and language ability. One of these children was a confirmed child who stuttered, and the other a confirmed typically fluent child. Byrd et al. (2015b) found that twelve out of the 14 bilingual SLPs “falsely identified the bilingual child who was a confirmed typically fluent speaker as a child who stutters” (i.e., 12 false positive identifications out of 14 cases), and “10 of the 14 correctly identified the bilingual child who stutters as such” (i.e., four false negatives). These results suggest that bilingual children are at risk of both overdiagnosis and underdiagnosis.

Byrd (2018) points to misconceptions about bilingualism on the part of clinicians as an additional factor that may increase the potential for overdiagnosis: Byrd et al. (2016) found that just over 20% of their sample of 207 trained Speech Language Pathologists considered bilingualism to be “a risk factor for either the onset of stuttering, the persistence of stuttering, or both”. A factor that may increase the risk of *under*diagnosis of stuttering in some bilingual children, also pointed out in Byrd (2018), is that parents of bilingual children may be accustomed to disfluencies in their children's speech, possibly lowering levels of parental concern and diminishing the reliability of parent concern as a “red flag”.

### The method for collecting information about stuttering used in the ECLS

The ECLS data are collected and maintained by the U.S. Department of Education's National Center for Education Statistics (NCES; http://nces.ed.gov/ECLS). The survey includes responses from parents, children, teachers, school administrators, and before- and after-school care providers throughout the elementary school years from Kindergarten through fifth grade. Parent interviews were administered by NCES field staff, “in most cases” by telephone, according to Tourangeau et al. (2015b). All questionnaire items were translated into Spanish before data collection began, and the interviews were administered by Spanish/English bilingual interviewers “if parent respondents preferred to speak in Spanish”, according to Tourangeau et al. (2019). Interviews were completed with parents who preferred to speak languages other than English or Spanish “by using an interpreter who translated the English version during the interview”.

In spring of the Kindergarten, first, and second grade, field staff asked “Did or does CHILD have any of the following? (...) A problem with stuttering.” Starting in the third grade round of data collection, the wording of the question was “Since last spring, has CHILD had a problem with stuttering?”. No further explanation was provided as to what was meant by “stuttering” or “problem with stuttering” in the Kindergarten, first or second grade parent interview text. In the third, fourth, and fifth grade rounds of data collection, “help texts,” visible to interview field staff, were included, as mentioned above. These were not read to parents, unless the parent asked the interviewer for additional clarification. It is unknown how many parents requested the explanation.

## Data and methods

### The data base

The current study is based on the complete set of public-use files of the Early Childhood Longitudinal Study, K Class of 2010-11 (Tourangeau et al., 2019). The publicly available data make it possible “track” cases longitudinally, by means of anonymous codes uniquely identifying each child.

The data base includes 18,174 children, including 8,847 (48.7%) girls, 9,288 (51.3%) boys, and 39 children whose parents declined to state the child's sex. The data set, when appropriately weighted, is intended to be a nationally representative sample.

### Variables considered

In addition to the question about the child's sex, we analyzed responses to the question asking parents about stuttering. The exact wording of that question is repeated below. In addition, we took into account two ECLS questions pertaining to the use of languages other than English in the children's home. Parents were first asked whether any language other than English was regularly spoken in the home. Parents who answered “yes” to that question were asked whether English was also spoken in the home.

The ECLS distinguishes three ways of identifying “language minority children”: (1) The group including all children in whose homes a language other than English is regularly used, (2) The subset in whose homes English is not regularly spoken, and (3) The subset classified as English Language Learners (ELLs), based on a combination of information from the children's schools and teachers, and English proficiency tests. Here, we focus on the first two groups, i.e., the group including all children in whose homes a language other than English was regularly spoken and the subset in whose homes English was not regularly used alongside a language other than English.

The first time the question about stuttering was asked was in Spring of the Kindergarten year. At that point, all of the children had been exposed to English for at least one semester and were probably bilingual to some degree. In fact, Najarian et al. (2019) note that “[b]y the spring of first grade, nearly all children demonstrated sufficient English proficiency to be assessed in English” in the portion of the ECLS that is administered to children, rather than their caregivers or teachers. In what follows, we therefore refer to the children in whose homes a language other than English was regularly used as the “bilingual” group, and to the subset in whose homes a language other than English was used exclusively as the “LOTE” (language other than English) subset.

The questionnaire items were worded as follows:

1. Did or does CHILD have any of the following? A problem with stuttering.
2. Is any language other than English regularly spoken in your home?
3. Is English also spoken in your home?

The names of variables coding responses to these questions contain the strings STUTER (sic), ANYLNG, and ENGTOO, respectively. The questions were asked across multiple grades. Following the ECLS nomenclature, the variables holding the responses were prefixed by the letter “P” for “parent” and a number ranging from 1 to 9, indicating the iteration of the test, from Fall of Kindergarten (1) to Spring of Fifth grade (9). For example, the variable “P2STUTER” in the “STUTER” group of variables holds the parent's response to the question about stuttering in Spring of the child's Kindergarten year.

Not all of the questions were asked every year. Table 1 shows the number of cases for which responses to the ECLS questionnaire item about stuttering and about home languages were available. The number of parents who were asked the question about stuttering decreases sharply over the years, from 13,046 in Kindergarten to 637 in fifth grade; in part, the decrease reflects a dependency that was introduced in the third-grade round of data collection: Starting in that grade, only those parents were asked about stuttering who had indicated that their child “pronounce[d] words, communicate[d] with and [understood] others slightly less well” or “much less well,” compared to other children of the same age, in response to a separate question during that same round of data collection. This dependency between the parents' response to the question about communication ability and being asked about stuttering is apparent in the full text of the parent interview. We return to this issue in the Discussion. Of the 13,046 parents who were asked about stuttering in K, only 2,442 were also asked about the use of languages other than English, of whom only 1,144 (those who responded “yes”) were additionally asked whether English was spoken alongside another language. Starting in grade 1, all parents who were asked about stuttering were also asked about the use of another language; a subset set of those families were also asked whether English was spoken alongside another language (2,109 in grade 1 and 154 in grade 5).

**Table 1:** Number of cases for which responses to the ECLS questionnaire item using the word “stuttering” were available for two questionnaire items about home languages: ANYLNG = “Is any language other than English regularly spoken in your home?”; ENGTOO = “Is English also spoken in your home?”.

**Grade**	**Stutter**	**Anylng**	**Engtoo**
K	13,046	2,442	1,144
1	6,879	6,879	2,109
2	n/a	n/a	n/a
3	823	823	n/a
4	792	n/a	n/a
5	637	637	154

In Kindergarten, many more parents were asked about stuttering (*n* = 13,046) than about the use of languages other than English. Starting in grade 1, all parents who were asked about stuttering were also asked about the use of other languages. The number of observations analyzed equals the number of available responses to ANYLNG (e.g., *n* = 2,442 for grade K) for analyses comparing monolingual English vs. bilingual groups, and to ENGTOO (e.g., *n* = 1,144 for grade K) for analyses of the two subsets of bilingual groups. Slight discrepancies between this and subsequent tables are due to *Don't know* and *Refused to state* responses, generally in the single digits.

As one can infer from these figures, different samples were taken for each of these questions over the years. Cases were added and subtracted each year, and relatively few parents were included during multiple consecutive years. The size of the overlap across years varies substantially. For example, responses to the question about stuttering were available for 4,848 cases in both K and 1, but only for 179 cases in grades 3 and 5. A mere six parents were asked about stuttering across all four grades where that question was asked (all grades except grade 2); one of these reported that their child stuttered from K to grade 5, and the other five responded ‘no'. Therefore, despite its longitudinal design, the database is ill-suited for tracking responses to the question about stuttering across grades for individual children.

The design of the ECLS is such that unweighted data are not intended to be representative of the population of children in grades K-5 in the U.S.. To compensate for geographical clustering and oversampling, observations need to be weighted (see e.g., Hahs-Vaughn, 2006; Hahs-Vaughn and Onwuegbuzie, 2006 for discussion). For the current study, we used the unweighted data, a point we return to below.

### Estimating the male/female ratio

The male/female ratio of stuttering is typically calculated by dividing the number of boys who stutter by the number of girls who stutter in some sample. For example, the male/female ratio in Månsson (2000) is reported as 1.65:1, on the basis that there were 33 boys who stuttered and 20 girls, and 33 divided by 20 is 1.65. That procedure is unproblematic if equal proportions of boys and girls are included. That was the case in Månsson (2000): Out of the 1,021 participants in that study (which included nearly all children born in the Danish island of Bornholm in the years 1990–1991), 51.8% were boys and 48.2% were girls.

When samples contain unequal numbers of boys and girls, the ratio of raw counts of children who stutter is driven in part by the proportion of boys and girls in the sample and is therefore less informative as a measure of the relative risk of stuttering among boys vs. girls. The gender balance in the ECLS varies substantially by grade and language background. For example, 64.0% (309 out of 483) of the children from homes where only English was spoken in grade 5 were boys, compared to 58.4% (90 out 154) of those in whose homes another language was spoken. To ensure that our estimates were not unduly affected by the imbalance in the sample, we therefore calculated two sets of male/female ratios: (1) the ratio of the numbers of boys and girls who were described as stuttering (i.e., the method employed, for example, in Månsson, 2000), and (2) a ratio controlling for the gender balance by grade, which we term the “prevalence ratio”. We estimated the prevalence ratio by dividing the percentage of boys described as stuttering, out of the boys in a given grade, by the percentage of girls described as stuttering, out of the number of girls in that grade. The resulting male/female ratio can be thought of as the expected ratio in a gender-balanced sample.

## Results

### **Q1** Does the proportion of children reported to stutter differ depending on whether another language besides English is spoken at the child's home?

We first asked whether the proportion of children reported to stutter differed depending on the child's home language background.

Table 2 shows the number of reported cases of stuttering and the reported prevalence for each grade for the children in whose homes only English was spoken (left-hand columns) vs. those in whose homes a language other than English was spoken, either alongside English or exclusively.

**Table 2:** Number and percentage of children reported to stutter, by grade and home language: homes in which English was the only language regularly used (“English only”, left-hand columns); homes in which a language other than English was regularly used (“Bilingual”, right-hand columns); “CNS” children not reported to stutter; “CWS” children reported to stutter; “%” percentage of children reported to stutter.

	**English only**			**Bilingual**		
**Grade**	**CNS**	**CWS**	**%**	**CNS**	**CWS**	**%**
K	1,233	61	4.7	1,071	67	5.9
1	4,652	115	2.4	2,019	77	3.7
3	551	69	11.10	159	33	17.2
5	441	40	8.3	129	22	14.6

The reported prevalence of stuttering among the children in whose homes English was the only language spoken ranged from 2.4% in first grade to 11.1% in grade 3. Among the bilingual children, the prevalence ranged from 3.7% in grade 1 to 17.2% in grade 3. In all grades for which this information was available, the reported prevalence was lower in the group of children in whose homes English was the only language spoken, compared to the corresponding grades for the children in whose homes a language other than English was spoken. In answer to **Q1**, then, we can say that the proportion of children reported to stutter certainly appeared to differ depending on whether another language besides English was spoken at the child's home.

Table 3 shows the reported prevalence for the two groups of bilingual children, i.e., the group in whose homes English was also spoken and the group in whose homes languages other than English were spoken exclusively. It will be noted that the sum of those two groups does not equal the total number of children in each grade whose parents responded “yes” when asked whether a language other than English was regularly used at the home. The reason for this is that responses to the question whether English was spoken alongside another language were entirely unavailable for grade 3 and only available for a subset of the parents who reported using a language other than English, as shown in Table 1. The reported prevalence of stuttering among the children in whose homes a language other than English was regularly spoken alongside English ranged from 3.4% in third grade to 14.9% in grade 5. Among the children in whose homes a language other than English was spoken exclusively, the prevalence was lowest (4.7% ) in grade K, increasing to 6.4% in grade 1. In grade 5, one out of the 10 children in that group reportedly stuttered.

**Table 3:** Children in whose homes a language other than English (LOTE) was spoken, either alongside English (left-hand columns) or exclusively (right-hand columns): “CNS” children not reported to stutter; “CWS” children reported to stutter; “Percent CWS” indicates the percentage of children reported to stutter.

	**English and LOTE regularly spoken**			**LOTE used exclusively**		
**Grade**	**CNS**	**CWS**	**% CWS**	**CNS**	**CWS**	**% CWS**
K	847	56	6.20	224	11	4.7
1	1,815	63	3.4	204	14	6.4
5	120	21	14.9	9	1	(10.0)

### **Q2** What is the male/female ratio among children who reportedly stutter? Is that ratio plausible, and does it depend on the child's home language?

Next, we turn to the male/female ratio of children reported to stutter. We ask whether the ratio is plausible in light of clinical research, and whether the extent to which it is plausible differs depending on the child's home language background.

Table 4 shows the number of boys and girls who were reported to stutter, as well as the male/female ratios, for the children in whose homes English was the only language regularly used, and for those in whose homes another language was used, either alongside English or exclusively.

**Table 4:** Number of children reported to stutter, by grade and sex, the male/female ratio, as the ratio of the number of boys and girls reported to stutter in each grade, and the “prevalence ratio”, as the ratio of the proportion of boys and the proportion of girls reported to stutter.

	**Only English used regularly**						**Languages other than English used regularly**					
	**Male**		**Female**				**Male**		**Female**			
**Grade**	**CNS**	**CWS**	**CNS**	**CWS**	**M/f ratio**	**Prev. ratio**	**CNS**	**CWS**	**CWS**	**CNS**	**M/f ratio**	**Prev. ratio**
K	634	47	599	14	3.36	3.00	524	40	547	27	1.48	1.51
1	2,109	87	2,543	28	3.11	3.64	921	50	1,098	27	1.85	2.12
3	341	50	210	19	2.63	1.54	94	23	65	10	2.30	1.48
5	276	32	165	8	4.00	2.26	75	14	54	8	1.75	1.22

In the monolingual English-speaking group, the male/female ratio is similar to typical estimates for teenagers and adults (~3:1 in grades K, 1, and 3, and 4:1 in grade 5). For the bilingual children, the male/female ratio was consistently lower, compared to the children in whose homes only English was spoken: The ratio ranged from 1.48 in K to 2.3 in grade 3. The “prevalence ratio” for each grade and language group, i.e., the proportion of boys reported to stutter divided by the proportion of girls reported to stutter, showed a similar pattern. Among those children in whose homes only English was spoken, boys reported to stutter substantially outnumbered girls reported to stutter in grades K, 1, and 5 (though not 3), by that metric, as well. The male/female ratio and prevalence ratio was low in all grades in the bilingual group, the male/female ratio ranging from 1.48:1 to 2.3:1, and the prevalence ratio ranging from 1.22 (in grade 5) to 2.12 (in grade 1). These ratios are low, compared to the usual accepted male/female ratio of 3:1 or 4:1. Neither group showed an increase in male/female ratio.

As we already saw in Table 1, responses to the question whether a language other than English was spoken alongside English vs. exclusively were only available for a subset of the families in whose homes a language other than English was spoken. The number of children in that subset who were reported to stutter was smaller still: There were 11 such children in K (7 boys, 4 girls), 14 in grade 1 (7 boys, 7 girls), and a single child in grade 5 (a girl). These numbers are so small that we refrained from calculating the male/female ratios.

### **Q3** Are the patterns of persistence of and recovery from (reported) stuttering consistent with clinical research?

As a further plausibility check of the data, we examine the reported onset and recovery, i.e., the times when individual children were reported to stutter, across multiple grades, taking advantage of the longitudinal design of the database.

As mentioned above, within-child information about stuttering across grades is sparse. However, the plausibility of the data can be gleaned to some degree by asking whether any children were reported to stutter in grades 1 and up who were not also reported to stutter in K. That was the case: Of the 192 parents who were asked about the use of a language other than English and who responded “yes” to the question about stuttering in grade 1, 80 had also been asked about stuttering in K. Of those 80, 44 had responded “no” to the question about stuttering at that time. Similarly, of the 62 children reportedly stuttering in grade 5, 48 had been asked about stuttering in K. Of those 48, 29 had responded “no” to the question about stuttering at that time. This means that a substantial portion of cases (about half of the cases that can be checked in this way) suggest that stuttering did not emerge until after grade K. Given the rarity of late onset of stuttering according to clinical research, these figures cast yet more doubt on the plausibility of the ECLS data as information about the actual disorder known as stuttering.

## Discussion

The ECLS K:2011, a longitudinal national survey of children across elementary school grades in the U.S., seemingly answers an urgent need for data about stuttering in bilingual children. At first glance, the information about stuttering in the ECLS seems to lend support to the claim that bilingualism might be a risk factor for stuttering: Within each grade for which information is available, the prevalence of (reported) stuttering is lower in the monolingual group, compared to bilingual groups. On closer inspection, however, it appears that the figures are implausible in light of widely accepted clinical research findings.

First, on the basis of clinical evidence, one would expect stuttering to be far more common in boys than in girls, with a male/female ratio of about 3:1 to 4:1 in the age group included in the ECLS. That was the case, by one measure at least, for the children from homes in which English was the only language spoken (ranging from 2.6:1 to 4.0:1); however, for children from homes where a language other than English was regularly used, the male/female ratio was below 2:1 in most grades, i.e., implausibly low, even when one corrects for the gender imbalance in the sample (cf. Table 4).

Secondly, clinical research suggests that onset and recovery occur before school entry in the vast majority of cases, and that recovery is more likely in girls than in boys (see e.g., Yairi and Ambrose, 2013; Bloodstein et al., 2021). Therefore, one would expect the male/female ratio either to remain stable over the elementary school years or to increase (due to cases of late recovery and/or a lag in the time when recovery is reported). That does not seem to be the case for the bilingual groups of children in the ECLS. Third, the pattern in the ECLS does not follow previous observations according to which the prevalence of stuttering gradually decreases with increasing distance from age of onset. Finally, there was an implausibly high number of children who were *not* reported to stutter in K, but who *were* described as stuttering in later grades.

In sum, the high prevalence, the low male/female ratio, the absence of an increase in that ratio across grades, the increase in overall prevalence across grades, and the implausibly high number of (apparent) “late-onset” cases are inconsistent with clinical reports of stuttering, casting doubt on whether the data in the ECLS are in fact information about stuttering.

Here, we discuss some possible reasons for the implausible patterns, the implications on research relying on parent reports of stuttering, and some of the limitations of the current study.

### What do the ECLS responses about stuttering reflect, if not stuttering?

If the data discussed here do not reflect cases of stuttering, then what do they reflect? We believe that one thing they reflect is the informal use of the word “stutter” to mean “speak disfluently or haltingly”. Criteria for stuttering identification have been the subject of intense debate (see e.g., Yairi, 2013). However, it is probably fair to say that researchers would expect parents to be given some form of explanation of what is meant by the word “stuttering”. That was not the case in the ECLS. Parents were not asked about formal diagnoses of stuttering by a speech-language pathologist, nor were they told that normal speech disfluencies do not constitute a disorder.

The fact that the question about stuttering in the ECLS is one of many questions asking about health conditions and disabilities might implicitly carry the message that what is being asked about is a health condition. However, the parent interviews touch on many facts not requiring formal diagnosis, such as the child's social skills, peer relationships, and school-liking.

It might be objected that many other health-related questions in the ECLS also did not make reference to formal diagnoses. What makes “stuttering” more worrisome as a questionnaire item than, say, “seizure” or “cerebral palsy” is that the word “stuttering” is often applied to phenomena that have nothing to do with the disorder known as stuttering.

We are not claiming that the informal use of the word “stuttering” would be applied exclusively to bilingual children, or that parents of monolingual children were more likely to interpret the question as a question about a disorder. Rather, we are speculating that parents' responses may reflect informal descriptions on the part of parents and teachers, and that the informal label may be used especially often when teachers (and parents) describe bilingual children's speech without intending to imply the presence of a disorder. Such descriptions may in turn lead their parents to respond “yes” when asked if their child had “a problem with stuttering”, which is the question they were asked in the ECLS.

The informal usage of the word “stuttering” and teachers' descriptions are only two factors that may have influenced parents' responses. Some additional considerations are differences in attitudes toward stuttering across cultural groups within a given host country (Üstün-Yavuz et al., 2021), toward proficiency in English, and toward the role of English vs. languages other than English in children's education.

The informal use of the word “stuttering” to describe disfluency generally (i.e., applied to monolingual and bilingual children alike), combined with its use to describe the speech of bilingual children with varying degrees of proficiency in English, could explain the high overall reported prevalence of “stuttering” in the ECLS, as well as the difference in male/female ratio in monolingual vs. bilingual children: Monolingual children may be described as “stuttering” frequently enough so as to drive up the reported prevalence, but infrequently enough so that the male/female ratio of actual cases of stuttering remains visible in the aggregated responses. For the bilingual children, the informal use of the word may be sufficiently common so as to mask the male/female ratio of actual cases of stuttering in bilingual children.

To explore the possibility that the informal use of the word “stuttering” was especially likely to be applied to bilingual children, we examined the data further, asking a question analogous to that in Howell et al. (2009). Among other questions, Howell et al. (2009) asked whether there was an elevated proportion of bilingual children among referrals to stuttering clinics. Analogously, we considered the children who were reported to stutter and ask what proportion came from homes where a language other than English was spoken. That question does not address whether bilingualism is a risk factor for stuttering. Rather, it amounts to asking whether the chances of being bilingual are higher among children described as stuttering. An analogy may help clarify the partial independence of these questions: The first question (“Is the level of reported stuttering elevated in bilingual children, compared to monolingual ones?”) is analogous to a food researcher asking whether an elevated proportion of meals described as “comfort food” by respondents to a survey are high-calorie meals, compared to the proportion of meals described as “comfort food” among low-calorie meals. The second question (“Are there more bilingual children among children described as stuttering than among children not described as stuttering?”) is analogous to asking whether there is an elevated percentage of high-calorie meals among those described as “comfort food”, compared to those not described as “comfort food”. The first is a question about the use of a particular informal description when describing high-calorie meals. The second is a question about the calorie content of meals described in a particular way.

As Table 5 shows, the percentage of bilingual children was indeed elevated among the children described as stuttering, compared to those not described in this way. This pattern was present in all grades for which this information was available. For example, children from homes in which a language other than English was spoken (the “bilingual” group) accounted for 52.3% of the children reported to stutter in grade K; among the children who were not reported to stutter, that percentage was 46.5%, i.e., lower by about 6%. In subsequent grades, the difference was larger, averaging about 11%. For example, in grade 1, the bilingual group accounted for 40% of the children reported to stutter, but only 30% of children not reported to stutter. We believe that this pattern reflects the informal use of the word “stuttering”, rather than any effect of bilingualism on the prevalence of stuttering.

**Table 5:** Number and percentage of children in whose homes English was the only language spoken (“En only”) and those in whose homes a language other than English was spoken (“bilingual”) among children described as stuttering vs. those not described as stuttering (right-hand columns).

	**Children reported to stutter**				**Children not reported to stutter**			
**Grade**	***n***	**En only**	**Bilingual**	**% bilingual**	***n***	**En only**	**Bilingual**	**% bilingual**
K	128	61	67	52.3	2,304	1,233	1,071	46.5
1	192	115	77	40.1	6,671	4,652	2,019	30.3
3	102	69	33	32.4	710	551	159	22.4
5	62	40	22	35.5	570	441	129	22.6

### Are the parents' responses “parent reports” of stuttering?

One might wonder whether the preceding discussion calls into question the validity of parent reports generally. We believe that the answer is “no”.

Parental report is an indispensable tool in stuttering research and clinical practice in part because of the episodic and intermittent nature of stuttering. As Franken et al. (2018) point out, parents may “underestimat[e] stuttering behaviors because parents grow accustomed to it”; and yet, reports from parents of children who do stutter have been found to be highly accurate, as Franken et al. (2018) also point out. For example, Yairi et al. (2005) (cited in Yairi and Ambrose, 2013) reported very close agreement between parent and clinician's identification of stuttering. Along similar lines, Einarsdóttir and Ingham (2009) compared parents of children who stuttered (CWS) vs. parents of children who did not stutter (CNS) vs. two highly experienced clinicians in terms of accuracy and consistency of identifying moments of stuttering. Einarsdóttir and Ingham (2009) found that parents of CWS were highly accurate, and more accurate than parents of CNS, in classifying disfluencies as stuttering vs. typical. Einarsdóttir and Ingham (2009) conclude that “parents are excellent judges of occurrences of stuttering in their own children.”

It will be noted that the selection of participants and the task in Einarsdóttir and Ingham (2009) differ in important respects from the ECLS: First, inclusion in the study required a clinical diagnosis and parental awareness of that diagnosis. This is typical for research relying on parental report, which usually involves children for whom a formal diagnosis of stuttering is under consideration or has already been made. Many parents of such children have extensive experience interacting with speech language pathologists. That is not the position most parents in the ECLS are in. Secondly, the task in Einarsdóttir and Ingham (2005) was to classify moments of disfluent speech as either stuttering symptoms vs. typical disfluencies. Reacting to a specific speech event is a different task from answering the question “Does your child stutter?”

In sum, we believe that the data in the ECLS do not reflect parent report in the sense in which the phrase is normally used in stuttering research. Our conclusions regarding the ECLS do not call parent reports into question.

### Limitations and future directions

One limitation of the current study is the use of unweighted data, and the variability in sample size across grades and groups of respondents. As a reviewer points out, the sample sizes were always larger for the monolingual group and in the lower grades, and prevalence estimates were always higher, the smaller the sample.

It is important to be clear on what types of conclusions one can and cannot draw, given the use of unweighted data in the current study, and given the substantial drop in sample size from K through 5th grade. Any inference to the population of as a whole (i.e., children in grades K through 5 in the U.S.) would be invalid, as would any inference based on models assuming random sampling and/or equality of variances across groups. As Hahs-Vaughn (2005) points out, “The results of analyses from unweighted samples cannot be generalized to any population other than that which was included in the original sample (i.e., the finite population)”.

However, the unweighted data do permit statements not requiring generalization to the population level or inferences relying on assumptions of parametric statistics. Several of the implausibilities we pointed out would persist even if the data were weighted. The children described as stuttering in K, then not stuttering, then stuttering again in a later grade are a case in point. Similarly, prevalence figures exceeding the lifetime incidence of stuttering are implausibly high, no matter the size of the subgroups for which the figures are claimed.

Other aspects of the analysis are suspect only if one posits a strong connection between stuttering and the groups considered in the design of the ECLS – much stronger than any research in the past decades has claimed, for example as strong as the association between maternal smoking and low birthweight. Korn and Graubard (1995) compared weighted and unweighted mean birthweights by mothers' smoking status, using data from the 1988 National Maternal and Infant Health Survey. Korn and Graubard (1995) point out that low-birthweight babies were oversampled, and that, as a consequence, the unweighted analysis overestimated the mean birthweight difference between children of mothers who did vs. did not smoke: Oversampling low-birthweight babies means that (1) mothers who smoked had a greater chance of being included, and as a consequence (2) the mean birthweight of babies of the group of mothers who smoked was lowered more than the mean in the non-smoking group. Tourangeau et al. (2019) note that Asians, Native Hawaiians, and Other Pacific Islanders (APIs) were oversampled in the base year of the ECLS. Oversampling of APIs would affect the prevalence estimates of stuttering if there were an association between API status and stuttering. Unlike maternal smoking and low birthweight, API status and stuttering are not known to be connected. Therefore, it is unclear what effect the oversampling of APIs would have on the estimated stuttering prevalence.

A different problem is posed by the shrinking sample sizes from K through 5th grade. One factor contributing to that pattern is implicit in two erratum notices (Tourangeau et al., 2015a, p. A-5; National Center for Education Statistics, 2024): the question about stuttering was part of a block of questions intended to be skipped in the first-grade round of data collection if it had already been asked during the Kindergarten round. This factor did not decrease the first-grade sample size as much as intended, however, because the question about stuttering was unintendedly asked a second time in over 4,800 cases (a similar error occurred in the second-grade round, not analyzed here). The respondents who were asked about stuttering in the first-grade round formed a subset of those who, during the Kindergarten round, had either (1) not been asked about the target child's communication abilities, or (2) been asked and had indicated at that time that the child “pronounce[d] words, communicate[d] with and [understood] others” at least as well as (or better than) other children their age, or (3) been asked and had chosen not to respond (*n* = 4) or responded with “I don't know” (*n* = 13) or, in one case, indicated that their child communicated slightly less well, compared to other children their age. These dependencies decreased the sample size and may, in addition, have lowered the percentage of children reported to stutter in the first-grade sample. Starting in third grade, respondents who were asked about stuttering formed a subset of those who indicated that their child “pronounce[d] words, communicate[d] with and [understood] others slightly less well” or “much less well,” compared to other children of the same age, i.e. the opposite of the constraint on the first-grade round. This restriction may or may not have inflated the percentage of children reported to stutter in third grade and up: In Kindergarten, children described as communicating at least as well as other children their age accounted for over two thirds of those whose parents responded “yes” to the question about stuttering (whose scope during that round included the years before school entry, making a direct comparison to the late elementary grades impossible). Therefore, it cannot be assumed that parents who described their children as communicating at least as well as other children their age would have responded “no,” had they been asked the question about stuttering. It is hoped that the current study may inspire follow-up investigations exploring suitable analytical options.

An additional issue that deserves further investigation concerns the language that the parent interviews were conducted in. As mentioned above, Spanish-language versions of the interviews were created ahead of data collection and were administered to parents who expressed a preference for speaking Spanish; parents who spoke languages other than Spanish or English were interviewed via live interpreters. We leave the possible effects of the language used during the interview as a matter of future research.

Finally, it is unclear whether being described as “stuttering” by parents or teachers actually increases the odds of referral for formal evaluation by speech language pathologists. There is some evidence suggesting otherwise: Morgan et al. (2016), for example, examined disparities in speech/language services to preschool-age children with expressive vocabulary delays. It was found that Black children and Hispanic from homes where a language other than English was spoken were less likely to receive speech/language services, compared to White children, when socioeconomic factors were brought under statistical control. The effects of overreferral (or underreferral) on formal diagnosis are also unclear. As mentioned earlier, there is evidence suggesting that some speech characteristics of bilingual children who do not stutter put them at risk of false positive diagnosis of stuttering (Byrd et al., 2015a). To our knowledge, it is currently unclear to what extent formal diagnoses, which take into account other factors besides disfluency counts (such as physical tension and the subjective experience of stuttering), are distorted by characteristics of bilingual children's speech generally.

## Conclusion

Previous research has pointed out the need for data on the prevalence of stuttering among bilingual children. We have argued that the data ostensibly about stuttering in a major longitudinal database (the ECLS:K-2011) fails to meet that need. The ECLS should not be taken at face value as information about the disorder known as stuttering. We believe that the responses to the ECLS questionnaire item that included the word “stuttering” likely reflects parents' and teachers' informal use of the word “stuttering”, rather than a disorder.

The question might arise, then, whether clinicians and researchers interested in stuttering should be concerned about the ECLS at all. We believe that they should be. The notion of bilingualism as a risk factor for stuttering, although considered to have been debunked by many researchers, is still with us. The matter is often characterized as an “open question”, even in the context of research showing alternative explanations for apparent higher rates of stuttering among bilingual populations. For example, Byrd (2018) state that “At present, there are insufficient data to support that such a risk exists.” Similarly, (Van Borsel, 2011, p. 266) states, correctly, that “[i]t is still a matter of debate whether or not bilingualism can cause stuttering”.

Research reports find their way into the media, often losing important nuance along the way. Research on bilingualism and stuttering is no exception. For example, Howell et al. (2009) suggested that early bilingualism (before the elementary school years) was a risk factor for stuttering and for stuttering persistence into the teenage years. Subsequent news reports read “Stutter risk for bilingual kids” (The Sydney Morning Herald, 2008), “Bilingual children more likely to stutter” (ScienceDaily, 2008), “Early bilingualism may increase stutter risk” (Australian Broadcasting Corporation, 2008). Howell et al.'s (2009) recommendation was for parents to raise their children monolingually in the parents' native language until school entry. That recommendation is mentioned in the body of some of the news reports, but never highlighted or included in a headline. None of the reports emphasize the value of parental languages or warn about potential adverse effects of learning the instructional languages of the school system before school entry (which are discussed, for example, in Fillmore, 2000). Reports linking bilingualism to stuttering can spread without sufficient nuance, to the detriment of multilingual families and society at large. Researchers have a responsibility, therefore, to proactively caution against the use of seemingly suitable, but actually misleading, sources of information.

## Data availability statement

Publicly available datasets were analyzed in this study. This dataset can be found at: https://nces.ed.gov/ecls/dataproducts.asp.

## Author contributions

SG was responsible for conception and design of the study, performing the analysis, as well as writing and revising all sections of the manuscript.

## References

[B1] Adams M. R. (1990). The demands and capacities model I: theoretical elaborations. J. Fluency Disord. 15, 135–141.

[B2] Andrews G. Hoddinott S. Craig A. Howie P. Feyer A.-M. Neilson M. (1983). Stuttering: a review of research findings and theories circa 1982. J. Speech Hear. Disord. 48, 226–246. 6353066 10.1044/jshd.4803.226

[B3] Australian Broadcasting Corporation (2008). Early Bilingualism May Increase Stutter Risk. Available online at: https://www.abc.net.au/news/2008-09-09/early-bilingualism-may-increase-stutter-risk/503922

[B4] Bloodstein O. Bernstein Ratner N. (2008). A Handbook of Stuttering. Clifton Park, NY: Thomson Delmar Learning.

[B5] Bloodstein O. Ratner N. B. Brundage S. B. (2021). A Handbook on Stuttering. San Diego, CA: Plural Publishing.

[B6] Boyle C. A. Boulet S. Schieve L. A. Cohen R. A. Blumberg S. J. Yeargin-Allsopp M.. (2011). Trends in the prevalence of developmental disabilities in US children, 1997–2008. Pediatrics 127, 1034–1042. 10.1542/peds.2010-2989 21606152

[B7] Briley P. M. Merlo S. (2020). Presence of allergies and their impact on sleep in children who stutter. Perspect. ASHA Spcl. Interest Groups 5, 1454–1466. 10.1044/2020_PERSP-20-00095

[B8] Brocklehurst P. H. (2013). Stuttering prevalence, incidence and recovery rates depend on how we define it: comment on Yairi & Ambrose'article Epidemiology of stuttering: 21st century advances. J. Fluency Disord. 38, 290–293. 10.1016/j.jfludis.2013.01.002 24238390

[B9] Brundage S. B. Corcoran T. Wu C. Sturgill C. (2016). Developing and using big data archives to quantify disfluency and stuttering in bilingual children. Semin. Speech Lang. 37, 117–127. 10.1055/s-0036-1580739 27111271

[B10] Brundage S. B. Ratner N. B. (2022). Linguistic aspects of stuttering: research updates on the language-fluency interface. Top. Lang. Disord. 42, 5. 10.1097/TLD.0000000000000269 35321534 PMC8936424

[B11] Byrd C. T. (2018). Assessing bilingual children: are their disfluencies indicative of stuttering or the by-product of navigating two languages? Semin. Speech Lang. 39, 324–332. 10.1055/s-0038-1667161 30142643

[B12] Byrd C. T. Bedore L. M. Ramos D. (2015a). The disfluent speech of bilingual Spanish–English children: considerations for differential diagnosis of stuttering. Lang. Speech Hear. Serv. Schl. 46, 30–43. 10.1044/2014_LSHSS-14-0010 25215876 PMC4689226

[B13] Byrd C. T. Haque A. N. Johnson K. (2016). Speech-language pathologists perception of bilingualism as a risk factor for stuttering. J. Commun. Disord. Deaf Stud. Hear. Aids. 4. 10.4172/2375-4427.1000158

[B14] Byrd C. T. Hoffman D. Gunderson E. (2015b). Identification of stuttering in bilingual Spanish-English-speaking children. Contemp. Issues Commun. Sci. Disord. 42, 72–87. 10.1044/cicsd_42_S_72

[B15] Byrd C. T. Werle D. Coalson G. A. Eggers K. (2020). Use of monolingual English guidelines to assess stuttering in bilingual speakers: a systematic review. J. Monolingual Bilingual Speech 2, 1–23. 10.1558/jmbs.12733

[B16] Cavenagh P. Costelloe S. Davis S. Howell P. (2015). Characteristics of young children close to the onset of stuttering. Commun. Disord. Q. 36, 162–171. 10.1177/1525740114549955

[B17] Centers for Disease Control and Prevention (2019). About the National Health Interview Survey.

[B18] Chaudhary C. Maruthy S. Guddattu V. Krishnan G. (2021). A systematic review on the role of language-related factors in the manifestation of stuttering in bilinguals. J. Fluency Disord. 68, 105829. 10.1016/j.jfludis.2021.105829 33556665

[B19] Choo A. L. Smith S. A. (2020). Bilingual children who stutter: convergence, gaps and directions for research. J. Fluency Disord. 63, 105741. 10.1016/j.jfludis.2019.105741 31883649

[B20] Choo A. L. Smith S. A. Li H. (2020). Associations between stuttering, comorbid conditions and executive function in children: a population-based study. BMC Psychol. 8, 113. 10.1186/s40359-020-00481-7 33129350 PMC7603732

[B21] Conradi E. (1912). Communications and discussions. Speech defects and intellectual progress. J. Educ. Psychol. 3, 35.

[B22] Craig A. Tran Y. (2005). The epidemiology of stuttering: the need for reliable estimates of prevalence and anxiety levels over the lifespan. Adv. Speech Lang. Pathol. 7, 41–46. 10.1080/14417040500055060

[B23] Einarsdóttir J. Ingham R. (2009). Accuracy of parent identification of stuttering occurrence. Int. J. Lang. Commun. Disord. 44, 847–863. 10.1080/13682820802389865 19105072

[B24] Einarsdóttir J. Ingham R. J. (2005). Have disfluency-type measures contributed to the understanding and treatment of developmental stuttering? Am. J. Speech Lang. Pathol. 14, 260–273. 10.1044/1058-0360(2005/026) 16396610

[B25] Fillmore L. W. (2000). Loss of family languages: should educators be concerned? Theory Pract. 39, 203–210. 10.1207/s15430421tip3904_3

[B26] Franken M.-C. J. Koenraads S. P. Holtmaat C. E. van der Schroeff M. P. (2018). Recovery from stuttering in preschool-age children: 9 year outcomes in a clinical population. J. Fluency Disord. 58, 35–46. 10.1016/j.jfludis.2018.09.003 30309634

[B27] Gahl S. (2020). Bilingualism as a purported risk factor for stuttering: a close look at a seminal study (Travis et al., 1937). J. Speech Lang. Hear. Res. 63, 3680–3684. 10.1044/2020_JSLHR-20-00364 32976049

[B28] Hahs-Vaughn D. L. (2005). A primer for using and understanding weights with national datasets. J. Exp. Educ. 73, 221–248. 10.3200/JEXE.73.3.221-248

[B29] Hahs-Vaughn D. L. (2006). Analysis of data from complex samples. Int. J. Res. Method Educ. 29, 165–183. 10.1080/17437270600891572

[B30] Hahs-Vaughn D. L. Onwuegbuzie A. J. (2006). Estimating and using propensity score analysis with complex samples. J. Exp. Educ. 75, 31–65. 10.3200/JEXE.75.1.31-65

[B31] Howell P. (2013). Screening school-aged children for risk of stuttering. J. Fluency Disord. 38, 102–123. 10.1016/j.jfludis.2012.09.002 23773664

[B32] Howell P. Davis S. Williams R. (2009). The effects of bilingualism on stuttering during late childhood. *Arch. Dis. Childhood* 94, 42–46. 10.1136/adc.2007.134114 18782846 PMC2597689

[B33] Iverach L. Menzies R. Jones M. O'Brian S. Packman A. Onslow M. (2015). Further development and validation of the unhelpful thoughts and beliefs about stuttering (UTBAS) scales: relationship to anxiety and social phobia among adults who stutter. Int. J. Lang. Commun. Disord. 46, 286–299. 10.3109/13682822.2010.495369 21575070

[B34] Karniol R. (1992). Stuttering out of bilingualism. First Lang. 12, 255–283.

[B35] Karniol R. (1995). Stuttering, language, and cognition: a review and a model of stuttering as suprasegmental sentence plan alignment (SPA). Psychol. Bull. 117, 104–124. 7870857 10.1037/0033-2909.117.1.104

[B36] Kohnert K. Medina A. (2009). “Bilingual children and communication disorders: a 30-year research retrospective,” in Seminars in Speech and Language 30, 219–233. 19851950 10.1055/s-0029-1241721

[B37] Korn E. L. Graubard B. I. (1995). Examples of differing weighted and unweighted estimates from a sample survey. Am. Stat. 49, 291–295.

[B38] Månsson H. (2000). Childhood stuttering: incidence and development. J. Fluency Disord. 25, 47–57. 10.1016/S0094-730X(99)00023-6

[B39] Mohammadi H. Nilipur R. Yadegari F. (2010). Prevalence of stuttering in Kurdish-Persian consecutive bilinguals in Iran. Asia Pac. J. Speech Lang. Hear. 13, 235–241. 10.1179/136132810805335001

[B40] Mohammadi H. Yadegari F. Nili-Pour R. Rahgozar M. A. (2008). Prevalence of stuttering in Javanroud's bilingual students. Arch. Rehabil. 9, 43–48.

[B41] Morgan P. L. Hammer C. S. Farkas G. Hillemeier M. M. Maczuga S. Cook M.. (2016). Who receives speech/language services by 5 years of age in the United States? Am. J. Speech Lang. Pathol. 25, 183–199. 10.1044/2015_AJSLP-14-0201 26579989 PMC4972004

[B42] Najarian M. Tourangeau K. Nord C. Wallner-Allen K. Vaden-Kiernan N. (2019). Early Childhood Longitudinal Study, Kindergarten Class of 2010–11 (ECLS-K:2011), Third-Grade, Fourth-Grade, and Fifth-Grade Psychometric Report (NCES 2020-123). Washington, DC: National Center for Education Statistics. Available online at: https://nces.ed.gov/pubsearch

[B43] National Center for Education Statistics (2024). ECLS Data Issues. U.S. Department of Education. Washington, DC: National Center for Education Statistics. Available online at: https://nces.ed.gov/ecls/dataissues.asp (accessed September 8, 2024).

[B44] OED Online (1989). Oxford English Dictionary Online. Available online at: www.oed.com/view/Entry/192285 (accessed May 18, 2023).

[B45] Riley G. Bakker K. (2009). SSI-4: Stuttering Severity Instrument. PRO-ED.

[B46] Rocha M. S. Yaruss J. S. Rato J. R. (2019). Temperament, executive functioning, and anxiety in school-age children who stutter. Front. Psychol. 10, 2244. 10.3389/fpsyg.2019.02244 31636587 PMC6788391

[B47] ScienceDaily (2008). Bilingual Children More Likely To Stutter. Available online at: https://www.sciencedaily.com/releases/2008/09/080908215938.htm

[B48] Smith A. Weber C. (2017). How stuttering develops: the multifactorial dynamic pathways theory. J. Speech Lang. Hear. Res. 60, 2483–2505. 10.1044/2017_JSLHR-S-16-0343 28837728 PMC5831617

[B49] Starkweather C. W. Gottwald S. R. (1990). The demands and capacities model II: clinical applications. J. Fluency Disord. 15, 143–157.

[B50] The Sydney Morning Herald (2008). Stutter Risk for Bilingual Kids.

[B51] Tichenor S. E. Yaruss J. S. (2019). Stuttering as defined by adults who stutter. J. Speech Lang. Hear. Res. 62, 4356–4369. 10.1044/2019_JSLHR-19-00137 31830837

[B52] Tourangeau K. Nord C. Lê T. Wallner-Allen K. Hagedorn M. C. Leggitt J.. (2015a). Early Childhood Longitudinal Study, Kindergarten Class of 2010–11 (ECLS-K:2011): User's Manual for the ECLS-K:2011 Kindergarten-First Grade Data File and Electronic Codebook, Public Version (NCES 2015-078). U.S. Department of Education. Washington, DC: National Center for Education Statistics. Available online at: https://nces.ed.gov/pubs2015/2015078.pdf

[B53] Tourangeau K. Nord C. Sorongon A. Hagedorn M. Daly P. Najarian M. (2015b). Early Childhood Longitudinal Study, Kindergarten Class of 2010-11 (ECLSK: 2011): User's Manual for the ECLS-K:2011 Kindergarten U.S. Department of Education. Washington, DC: National Center for Education Statistics. Available online at: https://nces.ed.gov/pubs2015/2015078.pdf

[B54] Tourangeau K. Nord C. Wallner-Allen K. Vaden-Kiernan N. Blaker L. Najarian M. (2019). Early Childhood Longitudinal Study, Kindergarten Class of 2010-11 (ECLS-K:2011): User's Manual for the ECLS-K:2011 Kindergarten-Fifth U.S. Department of Education. Washington, DC: National Center for Education Statistics. Available online at: https://nces.ed.gov/pubs2019/2019051.pdf

[B55] Travis L. E. Johnson W. Shover J. (1937). The relation of bilingualism to stuttering: a survey of the East Chicago, Indiana, schools. J. Speech Disord. 2, 185–189.

[B56] Üstün-Yavuz M. S. Warmington M. Gerlach H. St. Louis K. O. (2021). Cultural difference in attitudes towards stuttering among British, Arab and Chinese students: considering home and host cultures. Int. J. Lang. Commun. Disord. 56, 609–619. 10.1111/1460-6984.12617 33818900

[B57] Van Borsel J. (2011). Review of research on the relationship between bilingualism and stuttering. Multilingual Aspects Fluency Disord. 5, 247–270. 10.21832/9781847693570-013

[B58] Van Borsel J. Maes E. Foulon S. (2001). Stuttering and bilingualism: a review. J. Fluency Disord. 26, 179–205. 10.1016/S0094-730X(01)00098-5

[B59] Walsh B. Christ S. Weber C. (2021). Exploring relationships among risk factors for persistence in early childhood stuttering. J. Speech Lang. Hear. Res. 64, 2909–2927. 10.1044/2021_JSLHR-21-00034 34260279 PMC8740747

[B60] West J. (2017). National longitudinal studies of kindergarten children: historical context and ongoing contributions. AERA Open 3, 1–18. 10.1177/2332858417701684

[B61] Yairi E. (2013). Defining stuttering for research purposes. J. Fluency Disord. 38, 294–298. 10.1016/j.jfludis.2013.05.001 24238391

[B62] Yairi E. Ambrose N. (2013). Epidemiology of stuttering: 21st century advances. J. Fluency Disord. 38, 66–87. 10.1016/j.jfludis.2012.11.002 23773662 PMC3687212

[B63] Yairi E. Ambrose N. G. (1999). Early childhood stuttering I: persistency and recovery rates. J. Speech Lang. Hear. Res. 42, 1097–1112. 10515508 10.1044/jslhr.4205.1097

[B64] Yairi E. Ambrose N. G. Grinager N. (2005). Early Childhood Stuttering. Austin: Pro-ed.

[B65] Yaruss J. S. Quesal R. W. (2008). OASES: Overall Assessment of the Speaker's Experience of Stuttering Interpretive Report. Bloomington, MN: Pearson.

[B66] Zablotsky B. Black L. I. Maenner M. J. Schieve L. A. Danielson M. L. Bitsko R. H.. (2019). Prevalence and trends of developmental disabilities among children in the United States: 2009–2017. Pediatrics 144:e20190811. 10.1542/peds.2019-0811 31558576 PMC7076808

