# Corrigendum: Bilingualism as a risk factor for false reports of stuttering in the Early Childhood Longitudinal Study (ECLS-K:2011)

**DOI:** 10.3389/fpsyg.2024.1481398

**Published:** 2025-01-07

**Authors:** Susanne Gahl

**Affiliations:** Department of Linguistics, University of California at Berkeley, Berkeley, CA, United States

**Keywords:** stuttering, bilingualism, school-age children, linguistic minorities, parent report, epidemiology, age factors, sex factors

In the published article, there was an error. The article erroneously stated that no definition of stuttering was provided in the ECLS survey process. Starting in the third grade round of data collection, there was a definition of stuttering available to interview field staff.

A correction has been made to **Introduction**, **Paragraph Number 7**. This sentence previously stated:

No clarification was provided in the ECLS survey process as to whether the word “stuttering” in the ECLS was intended as the name of a disorder.

The corrected sentence appears below:

No clarification was included in the question as to whether the word “stuttering” in the ECLS was intended as the name of a disorder. Starting in the third-grade round of data collection, a “help text” was available for interviewers to read to parents upon request. The help text read “*A Stuttering problem refers to difficulty producing fluent (flowing or effortless) speech. The child cannot speak sentences or groups of words with ease. Instead, the child hesitates before saying some words. They may seem as if they are mentally blocking on a word. This hesitation makes it hard for the listener to understand what the child is trying to say*.” That explanation falls short in several respects: Most children who stutter produce sentences or groups of words with ease some of the time. Most importantly, the help text fails to differentiate stuttering from normal disfluencies, tip-of-the-tongue states, or other speech hesitations not due to stuttering, and it falsely implies that symptoms of stuttering generally impede intelligibility.

In the published article, there was an error. The article correctly reported the wording of the question about stuttering as asked during the Kindergarten, first, and second grade rounds of data collection; however, starting in the third grade round of data collection, the wording of the question was changed so as to restrict the temporal scope to the immediately preceding year.

A correction has been made to **Background**, ***The method for collecting information*** ***about stuttering used in the ECLS***, **Paragraph 2**. This sentence previously stated:

During each interview that included the question about stuttering, field staff asked “Did or does CHILD have any of the following? (...) A problem with stuttering.” No further explanation was provided as to what was meant by “stuttering” or “problem with stuttering”.

The corrected sentence appears below:

In spring of the Kindergarten, first, and second grade, field staff asked “Did or does CHILD have any of the following? (...) A problem with stuttering.” Starting in the third grade round of data collection, the wording of the question was “Since last spring, has CHILD had a problem with stuttering?”. No further explanation was provided as to what was meant by “stuttering” or “problem with stuttering” in the Kindergarten, first or second grade parent interview text. In the third, fourth, and fifth grade rounds of data collection, “help texts,” visible to interview field staff, were included, as mentioned above. These were not read to parents, unless the parent asked the interviewer for additional clarification. It is unknown how many parents requested the explanation.

In the published article, there was an error. The article failed to note that there were constraints on which parents were asked about stuttering following the Kindergarten round of data collection, both by design (starting in the third-grade round) and (prior to that) consequent to a data collection anomaly noted in user manual for grade 1 of the ECLS-K:2011 (Tourangeau et al., 2015a) and a recently-released correction notice (National Center for Education Statistics, 2024).

A correction has been made to **Data and Methods**, ***Variables considered***, **Paragraph 7**. This sentence previously stated:

The number of parents who were asked the question about stuttering decreases sharply over the years, from 13,046 in Kindergarten to 637 in fifth grade.

The corrected sentence appears below:

The number of parents who were asked the question about stuttering decreases sharply over the years, from 13,046 in Kindergarten to 637 in fifth grade; in part, the decrease reflects a dependency that was introduced in the third-grade round of data collection: Starting in that grade, only those parents were asked about stuttering who had indicated that their child “pronounce[d] words, communicate[d] with and [understood] others slightly less well” or “much less well,” compared to other children of the same age, in response to a separate question during that same round of data collection. This dependency between the parents' response to the question about communication ability and being asked about stuttering is apparent in the full text of the parent interview. We return to this issue in the Discussion.

In the published article, there was an error. The article did not include a discussion of constraints on which parents were asked about stuttering following the Kindergarten round of data collection.

A correction has been made to **Discussion**, ***Limitations and future directions***, **Paragraph 5**. This sentence previously stated:

A different problem is posed by the shrinking sample size from K through 5th grade. That issue has the potential to distort the within-group estimates, if it is the case that parents who responded ‘yes' to the question about stuttering were disproportionately likely to be retained in the sample. Further investigation is needed to determine whether that was the case, and whether either weighting or imputation yield plausible results when applied to the current data set.

The corrected sentence appears below:

A different problem is posed by the shrinking sample sizes from K through 5th grade. One factor contributing to that pattern is implicit in two erratum notices (Tourangeau et al., 2015a, p. A-5; National Center for Education Statistics, 2024): the question about stuttering was part of a block of questions intended to be skipped in the first-grade round of data collection if it had already been asked during the Kindergarten round. This factor did not decrease the first-grade sample size as much as intended, however, because the question about stuttering was unintendedly asked a second time in over 4,800 cases (a similar error occurred in the second-grade round, not analyzed here). The respondents who were asked about stuttering in the first-grade round formed a subset of those who, during the Kindergarten round, had either (1) not been asked about the target child's communication abilities, or (2) been asked and had indicated at that time that the child “pronounce[d] words, communicate[d] with and [understood] others” at least as well as (or better than) other children their age, or (3) been asked and had chosen not to respond (*n* = 4) or responded with “I don't know” (*n* = 13) or, in one case, indicated that their child communicated slightly less well, compared to other children their age. These dependencies decreased the sample size and may, in addition, have lowered the percentage of children reported to stutter in the first-grade sample. Starting in third grade, respondents who were asked about stuttering formed a subset of those who indicated that their child “pronounce[d] words, communicate[d] with and [understood] others slightly less well” or “much less well,” compared to other children of the same age, i.e. the opposite of the constraint on the first-grade round. This restriction may or may not have inflated the percentage of children reported to stutter in third grade and up: In Kindergarten, children described as communicating at least as well as other children their age accounted for over two thirds of those whose parents responded “yes” to the question about stuttering (whose scope during that round included the years before school entry, making a direct comparison to the late elementary grades impossible). Therefore, it cannot be assumed that parents who described their children as communicating at least as well as other children their age would have responded “no,” had they been asked the question about stuttering.

The authors apologize for this error and state that this does not change the scientific conclusions of the article in any way. The original article has been updated.
